# The Whole-Genome Sequence of Plasmid-Bearing Staphylococcus argenteus Strain B3-25B from Retail Beef Liver Encodes the Type VII Secretion System and Several Virulence Factors

**DOI:** 10.1128/MRA.00962-19

**Published:** 2019-11-07

**Authors:** Leena Neyaz, Anand B. Karki, Mohamed K. Fakhr

**Affiliations:** a Department of Biological Science, The University of Tulsa, Tulsa, Oklahoma, USA; Broad Institute

## Abstract

The whole-genome sequence of Staphylococcus argenteus strain B3-25B, isolated from retail beef liver, comprises a circular chromosome (2,676,222 bp) and a single plasmid (21,570 bp). The chromosome harbors genes encoding the type VII secretion system and several virulence factors.

## ANNOUNCEMENT

Staphylococcus argenteus belongs to the Staphylococcus aureus-related complex, formerly known as Staphylococcus aureus clonal complex 75 (1). Recent studies revealed the global distribution of *S. argenteus* isolated from both humans and animals (2–4); however, its prevalence and distribution remain unclear due to the difficulties in differentiating between methicillin-resistant S. aureus (MRSA) and *S. argenteus* (5). Initially, *S. argenteus* was thought to be less virulent than S. aureus due to the lack of staphyloxanthin, but subsequent deaths have shown its clinical importance (2, 6, 7). Moreover, genes encoding toxins such as staphylococcal enterotoxin, hemolysin, and Panton-Valentine leukocidin were associated with *S. argenteus* (4, 7–9). A recent study indicated the ability of *S. argenteus* to cause staphylococcal food poisoning, which indicates that this bacterium is highly toxigenic and is a potential foodborne pathogen (10).

Herein, we announce the complete genome sequence of *S. argenteus* strain B3-25B, which comprises a single chromosome and plasmid (pSALNBL21) of 2,676,222 bp and 21,570 bp, respectively. *S. argenteus* strain B3-25B was previously isolated in our laboratory from retail beef liver (11). *S. argenteus* cells were grown in tryptic soy agar (TSA) at 37°C for 16 to 24 h and then used for genomic DNA isolation. Genomic DNA was isolated using a DNeasy blood and tissue kit (Qiagen, Valencia, CA). Plasmid DNA was confirmed using S1 nuclease pulsed-field gel electrophoresis (PFGE), as described previously (12). A library was prepared with the Nextera XT kit (Illumina, Inc., San Diego, CA), and next-generation sequencing was conducted using an Illumina V2 reagent kit with 2 × 250 cycles. The sequence reads which passed the initial MiSeq quality standards were assessed using the CLC Genomics Workbench v. 12.0 (Qiagen) and trimmed for adapters, low-quality reads, and short reads. A total of 2,988,566 paired-end reads were submitted to the comprehensive genome analysis service at PATRIC v. 3.5.39 (13) for *de novo* assembly using the default settings of SPAdes v. 3.13.0 (14). A total of 39 contigs were joined to assemble the circular chromosome by aligning them against the closest reference, Staphylococcus argenteus strain BN75 (NCBI RefSeq accession number NZ_CP015758), using the default parameters of the genome-finishing module plugin v. 1.9 of the CLC Genomics Workbench v. 12.0 (Qiagen). The reads were also submitted to the plasmidSPAdes tool using default settings (15), which resulted in the identification of the plasmid as a single circular contig.

The B3-25B genome was annotated using the RAST toolkit (RAST*tk*) (16) and the NCBI Prokaryotic Genome Annotation Pipeline. The chromosome has a GC content of 32.3% and contained 2,447 predicted coding DNA sequences (CDSs), including 63 RNAs and 287 subsystems. Chromosomally borne virulence factors included autolysin-, fibronectin-, and fibrinogen-binding proteins, extracellular and intercellular adherence proteins, elastin-binding proteins, and staphylococcal protein A. Genes encoding protease, hyaluronate lyase, lipase, staphylocoagulase, and thermonuclease were identified, along with genes involved in immune system invasion and the type VII secretion system. Of special interest was the presence of genes encoding hemolysin (*hly*, *hld*, *hlgABC*), exfoliative toxin, and exotoxins 2, 7, 9, 10, 11, 12, 13, 15, 16, and 23. Genetic elements that foster survival during food processing were detected, such as those that enhance survival in response to electrophiles, osmotic and oxidative stress, and thermal stress. Genes responsible for heavy metal tolerance (cobalt/zinc/cadmium, arsenate, and mercury), antibiotic resistance (fluoroquinolones and multidrug resistance efflux pumps), and bile resistance were identified. The *blaZ* gene was detected on the plasmid, which may confer resistance to β-lactam antibiotics.

### Data availability.

The whole-genome shotgun data for Staphylococcus argenteus strain B3-25B have been deposited in GenBank under the accession numbers CP042286 for the chromosome and CP042287 for plasmid pSALNBL21. The raw sequence reads have been deposited in the Sequence Read Archive (SRA) under accession number PRJNA555633.
